# Occurrence of Antibiotic Resistance Genes and Bacterial Markers in a Tropical River Receiving Hospital and Urban Wastewaters

**DOI:** 10.1371/journal.pone.0149211

**Published:** 2016-02-24

**Authors:** Naresh Devarajan, Amandine Laffite, Crispin Kyela Mulaji, Jean-Paul Otamonga, Pius Tshimankinda Mpiana, Josué Ilunga Mubedi, Kandasamy Prabakar, Bastiaan Willem Ibelings, John Poté

**Affiliations:** 1 University of Geneva, Institute F. A. Forel and Institute of Environmental Sciences, Geneva, Switzerland; 2 University of Kinshasa (UNIKIN), Faculty of Science, Department of Chemistry, Kinshasa XI, Democratic Republic of the Congo; 3 Université Pédagogique Nationale (UPN), Croisement Route de Matadi et Avenue de la Libération, Quartier Binza/UPN, Kinshasa, Democratic Republic of the Congo; 4 Postgraduate and Research Department of Zoology, Jamal Mohamed College, Tiruchirappalli-620020, Tamil Nadu, India; Nankai University, CHINA

## Abstract

The occurrence of emerging biological contaminants including antibiotic resistance genes (ARGs) and Faecal Indicator Bacteria (FIB) is still little investigated in developing countries under tropical conditions. In this study, the total bacterial load, the abundance of FIB (*E*. *coli* and *Enterococcus* spp. (ENT)), *Pseudomonas* spp. and ARGs (*bla*_TEM_, *bla*_CTX-M_, *bla*_SHV_, *bla*_NDM_ and *aadA*) were quantified using quantitative PCR in the total DNA extracted from the sediments recovered from hospital outlet pipes (HOP) and the Cauvery River Basin (CRB), Tiruchirappalli, Tamil Nadu, India. The abundance of bacterial marker genes were 120, 104 and 89 fold higher for the *E*. *coli*, *Enterococcus* spp. and *Pseudomonas* spp., respectively at HOP when compared with CRB. The ARGs *aadA* and *bla*_TEM_ were most frequently detected in higher concentration than other ARGs at all the sampling sites. The ARGs *bla*_SHV_ and *bla*_NDM_ were identified in CRB sediments contaminated by hospital and urban wastewaters. The ARGs abundance strongly correlated (r ≥ 0.36, *p* < 0.05, n = 45) with total bacterial load and *E*. *coli* in the sediments, indicating a common origin and extant source of contamination. Tropical aquatic ecosystems receiving wastewaters can act as reservoir of ARGs, which could potentially be transferred to susceptible bacterial pathogens at these sites.

## Introduction

Antibiotics (Abs) are extensively used in prophylaxis of human and veterinary medicine. A number of Abs administrated to human/animals are partially metabolized in the digestive track and discharged in the hospital/communal effluents, which end up in environmental water bodies, either treated/untreated [1]. Antibiotic resistant bacteria (ARB) and antibiotic resistant genes (ARGs) from human/animal sources, along with excessive use of Abs in the human, veterinary and agricultural settings are currently considered as a serious environmental problem [1] Antibiotic resistance (AR) is a growing global public threat with serious health, political and economic implications. New forms of AR could spread with ease across international boundaries and between continents [2, 3]. In the environment the increased dissemination of ARB is possibly caused by mechanisms involving horizontal gene transfer (HGT) of ARGs, selective pressure induced by various contaminants (Abs, metals, biocides) in the environment, gene mutation and recombination [4]. In recent years, research studies outside the clinical settings on AR have begun to receive attention. Understanding the diversity patterns of antibiotic resistance genes and biological role of AR mechanisms will help to control its threats to human/animal health [5].

In many parts of the globe freshwater resources polluted by microbial contaminants is still a major problem [6]. Faecal indicator bacteria (FIB) (*Escherichia coli* (*E*. *coli*) and *Enterococcus* spp., (ENT)) residing in the gastrointestinal tract of warm blooded animals are generally used to monitor the microbial quality of water sources. Additionally, polluted surface waters and sediments can contain a variety of pathogenic microbes including bacteria, viruses and protozoa [6, 7]. The choice of bacterial indicators is thus very important. Bacteria belonging to the *Pseudomonas* genus are extensively present in the environment, such as water soil and sediment. Being known for its innate resistance mechanisms, *Pseudomonas* spp. are capable of staying viable in the aquatic environment for long periods [8], which carries the hazard of spreading ARGs and mobile genetic elements and can cause infections in humans [8, 9]. Sediments may contain 100–1000 fold higher bacterial counts than the overlying water [10]. Estimation of microbial contaminants in sediments serves as a stable index for long-term water quality risks [6, 7, 10–12]. Hence, sediments provide us with the opportunity to address the persistence of microbial contaminants/ARGs and the potential impact of the emergence of resistant bacteria from wastewaters and its transfer to the freshwater microbial community.

Effluents from hospital, industrial waste, domestic sewage, and urban/agricultural runoff in developing countries represent a significant source of emerging contaminants (metals, ARGs, ARB) for the receiving environment as the effluents are discharged to the sewer systems, rivers, lakes, and seas without prior treatment, which then may accumulate in the sediments [7, 8, 13]. Rivers and lakes are considered as putative reservoirs of emerging contaminants (drugs, metals, ARGs), since they collect wastewaters containing various contaminants from different origins [1, 14, 15]. Studies investigating rivers as a potential reservoirs of ARGs are still very limited in tropical areas, including South India [16]. However, a few of such studies have reported the prevalence of clinically relevant ARGs in environmental samples from North India [17, 18].

The presence of emerging pollutants such as ARB/ARGs, metals in wastewaters and their dissemination to the environmental compartment have become a topics of scientific interest over the last few decades [11, 19, 20]. In developing countries (under tropical conditions). A few studies [13, 21] reported on the physical-chemical characteristics of sediments receiving untreated hospital and urban effluent waters, however, little information is available on the development of microbial contaminants in receiving water systems. Hence there is a paucity of information regarding the microbial water quality in the tropics tropical rivers as well as about the prevalence of clinically relevant ARGs resulting from untreated urban and hospital effluents. The aim of the research presented in this study is to assess the role of untreated hospital and urban wastewaters on the accumulation of microbial contaminants in the sediment of receiving systems in the Cauvery River Basin, Tiruchirappalli, Tamil Nadu, India. This assessment was based on quantitative polymerase chain reaction (qPCR) on ARGs (*bla*_TEM_, *bla*_CTX-M_, *bla*_SHV_, *bla*_NDM_ and *aadA*), total bacterial load, and selected bacterial marker genes FIB and *Pseudomonas* spp. ARGs selected for this study were based on the following criteria: (i) clinically relevant genes (human risk); (ii) genes conferring resistance to frequently used Abs (eg: penicillins and aminoglycosides) and newer extended-spectrum ß-lactams (carbapenems and cephalosporins); (iii) ARGs previously reported in mobile genetic elements; and (iv) prevalence of metallo-ß-lactamases (*bla*_NDM_) in South India [11, 17, 18]. This research presents useful tools for the evaluation of emerging microbial contaminants in tropical aquatic ecosystems which can be applied in similar environments.

## Materials and Methods

### Study area and sample collection

This study was carried out in the Cauvery River Basin (Tiruchirappalli, Tamil Nadu, India) [13, 21]. Cauvery River, located in the southern part of the Indian sub-continent is estimated to cover a drainage area of 81,555 km^2^, with many tributaries generally flowing to the south and east of the states of Karnataka and Tamil Nadu. The river flows between 75°27' to 79°54' east longitude and 10°9' to 13°30' north latitude. From Coorg in the Western Ghats to the river mouth at the Bay of Bengal, the river flows through a densely populated area. The climate is tropical and its main tributaries include Hemavati, Kabini, Bhavani and Amaravati. Cauvery River is primarily supplied by monsoon rains, and the discharge of this rain-fed river fluctuates seasonally. Cauvery River water is mostly used for irrigation, household consumption and the generation of electricity. The river, during its course, receives a substantial amount of industrial effluents, untreated municipal sewage, urban runoff and agricultural runoff [22, 23]. Located in the central part of Tamil Nadu, at a latitude of 10°10' and 11°20' north and a longitude of 78°10' and 29°0' east Tiruchirappalli has a tropical climate with ca. 0.75 million inhabitants as of 2001 and the recent estimates measures to be ca. 1 million. The city generates an average of 68 million liters day^-1^ of wastewater and collects 42 million liters day^-1^ in its existing sewage system with the end point being the nearby water sources (Cauvery River) after a partial treatment. At Tiruchirappalli, large industries (steel plants, distilleries and chemical factories, leather tanning and pesticides production) discharge their wastewaters to Uyakkondan canal. This irrigation canal serves as a major source of water for more than 32,000 acres of agricultural land, 36 tanks, and also serves as the major conduit for receiving the effluent waters from large industries. The sampling locations and the collection points for our study are identical to a previous study [13]. Hence the sampling site E1 (effluent discharge point) is free from wastewater from large industries, but does receive wastewater from domestic sources, hospitals and small-scale industries [13]. Briefly, the surface sediments (n = 45) were collected from: (1) five selected hospital outlet pipes (HOP), before being released into the municipal sewers, for a period of 4 months (June to September), during 2 calendar years (2012 and 2013) and (2) in the Cauvery River Basin (CRB),Tiruchirappalli, Tamil Nadu, India. The surface sediments (2–6 cm) in the CRB were collected at 5 specific points labelled as R0–5 km upstream of the effluent discharge point on the river as a control sample; E1 –effluent discharge point to the river; R1, R2, and R3 located respectively 5, 10 and 15 km downstream on the river from the E1 site. In CRB the sediments were retrieved from 1m water depth during September 2013. The sediments were collected in sterile plastic bags and stored at -20°C until they were shipped to Institute F.A. Forel, University of Geneva, Switzerland for further analysis. Seepage samples are not listed in the DGFT Notification number 27/2007 issued by the Government of India, Ministry of Commerce and Industry, Department of Commerce, and therefore no permit is required for export [18]. Sediment samples were collected from public areas and facilities.

### Total DNA extraction and Quantification of FIB and ARGs

The total DNA from sediment samples was extracted using the Ultraclean soil DNA Kit (Mo Bio Labs, Solana Beach, CA) according to the manufacturer’s recommendations. The isolated DNA concentration was measured using Qubit assay kit (Life Technologies, Switzerland) following the manufacturer’s instructions and stored at -20°C. For each sediment sample, DNA extraction was performed with three replicate samples (from the same sampling point) to take heterogeneity into account. The DNA samples were diluted to 10x and 50x to avoid inhibitors to the PCR reaction. Specific genes targeted included ARGs (*bla*_TEM_, *bla*_CTX-M_, *bla*_SHV_, *bla*_NDM_ and *aadA*), and selected bacterial marker genes {*E*. *coli* (*uidA* gene); ENT and *Pseudomonas* spp. (16S rRNA gene)}. The selection of ARGs conferring resistance to ß-lactams and aminoglycosides were based on criteria of clinical relevance, i.e. frequently used Abs, ARGs often reported to be present on mobile genetic elements [11] and to better understand the prevalence of metallo-ß-lactamases in Southern India. The total abundance of the16S rRNA gene was also quantified to estimate total bacterial population size and used for the normalization of ARGs and selected bacterial marker genes abundance relative to total bacterial community size. The primers, qPCR reactions (Table A in S1 file), control plasmids, calculation for absolute gene copy numbers (gene concentration) and the gene copy numbers normalized to 16S rRNA gene (abundance) were performed as described in previous study [11]. Briefly, all genes were quantified with KAPA SYBR^®^ FAST qPCR Master Mix Universal Kit (KAPA Biosystems, MA, USA) on Eco^™^ Real-time PCR system (Illumina, Switzerland) using the primers (Invitrogen, USA) for selected gene targets (Table A in S1 file). The assay was carried out in a final 10 μl reaction volume containing 2 μl of template DNA, 0.5 μl of each forward and reverse primer (10 pmol μl^-1^), 2 μl of nuclease free water and 5 μl of qPCR master mix. The qPCR program was initially held for 2 min at 50°C for UDG incubation, 10 min at 95°C for the polymerase activation; followed by 40 cycles of 95°C for 30 s, optimal Tm (Table A in S1 file) for 30s and 72°C for 30 s. Data were acquired at 72°C. A melt curve was run at the end for the presence of unique PCR reaction product. All reactions included a negative and a positive control (10 x dilutions of control plasmids). Samples were considered to be below the limit of detection (LOD) or negative for a target gene if ≥2 out of 3 technical replicates were negative or if sample Ct values were ≥Ct of negative controls. For each reaction, the efficiency of the assay was calculated by using the measured slope of the standard curve (E = 10^(−1/slope)^ − 1). A reaction was considered applicable if the slope (-3.1 to -3.6), efficiency (90–110%) and R^2^ (>0.99) values were within the recommended range.

### Data Analysis

All data were log-transformed to improve sample normality before analysis. A statistical treatment of data Correlation matrix (Pearson), Principle Component Analysis (PCA) and Agglomerative hierarchical clustering (AHC) computation based on Ward's method were performed on XLSTAT (Version 2015.1.03.15828 Addinsoft 1995–2014). Statistical significance was always defined by 95% confidence intervals (*p* < 0.05). Sediment total water content was measured by drying the sediment samples at 60°C for overnight and the weight loss was measured for the percentage of water content [7]. The percentage of water content was used to calculate for the dry sediment weight. The 16S rRNA gene (total bacterial load) and the selected marker genes and ARGs in the samples are expressed as “log transformed gene copy numbers” in per gram of dry sediment weight normalized to the DNA extraction yield. The “relative abundance” of the selected genetic marker genes (normalized to 16S rRNA gene) were emphasized by the ratio = (copy number of a gene) / (copy number of 16S rRNA gene) for each sample [11, 19].

## Results and Discussion

### Bacterial population quantification

**Fig 1:**
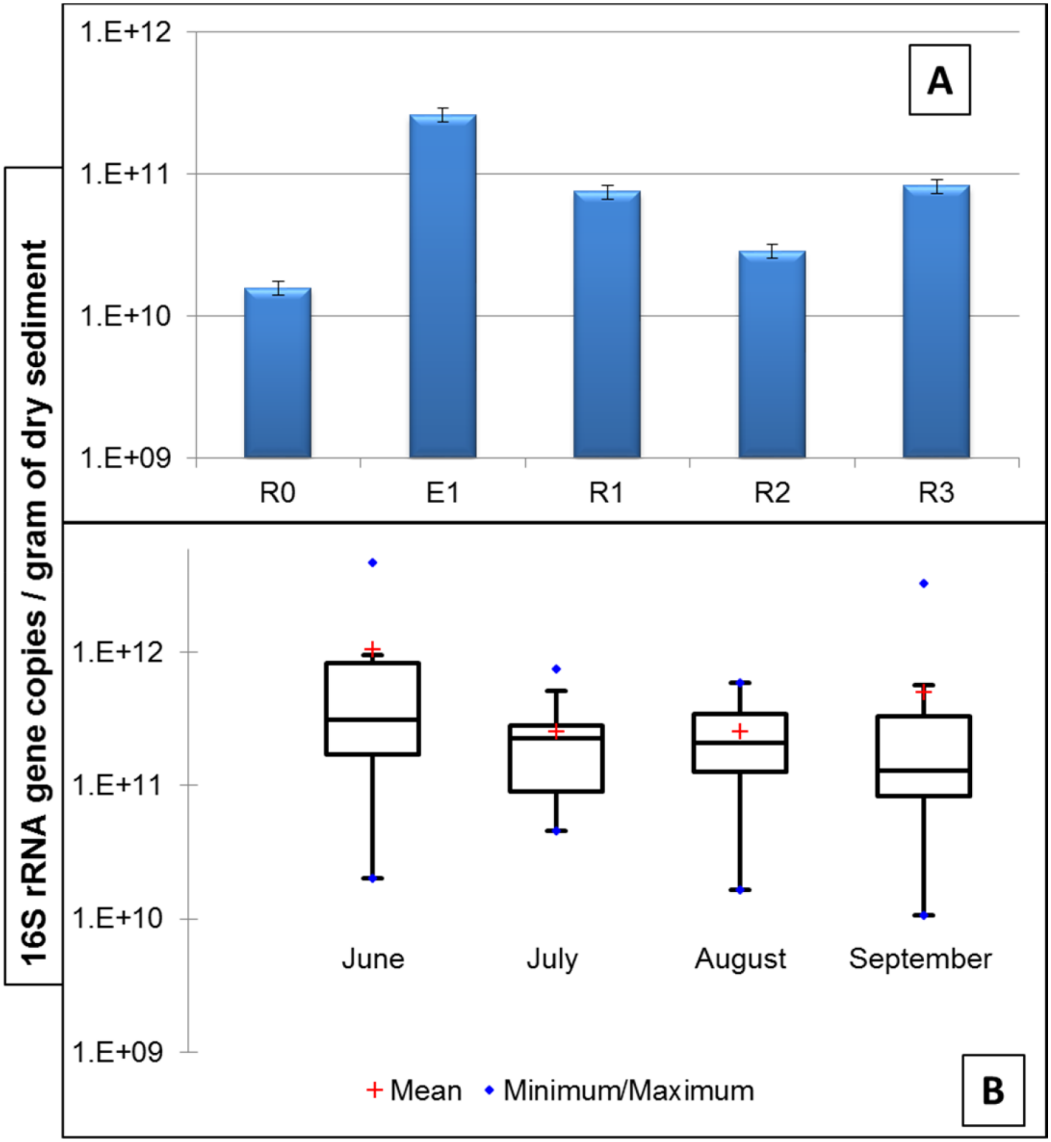
qPCR quantification of 16S rRNA gene in the surface sediment samples of CRB (A) and HOP (B). Values (log transformed) are expressed for the copy number per gram of sediment dry weight normalized to the DNA extraction yield. The box plot represents the first and third quartile, the blue dots on maximum/minimum values and the red cross indicate mean.

The total bacterial load in the sediment samples (16S rRNA gene copy numbers) wasin the range of 1.5 x 10^10^ to 2.6 x 10^11^ and 1.07 x 10^10^ to 4.7 x 10^12^ copy numbers g^−1^ of dry sediment collected from CRB and HOP, respectively (Fig 1). The total average bacterial load at the HOP was ca.82 fold higher when compared to CRB. In CRB, the total bacterial load after the effluent discharge point were 3.1 to 16.5 fold higher than control site (R0).

**Fig 2:**
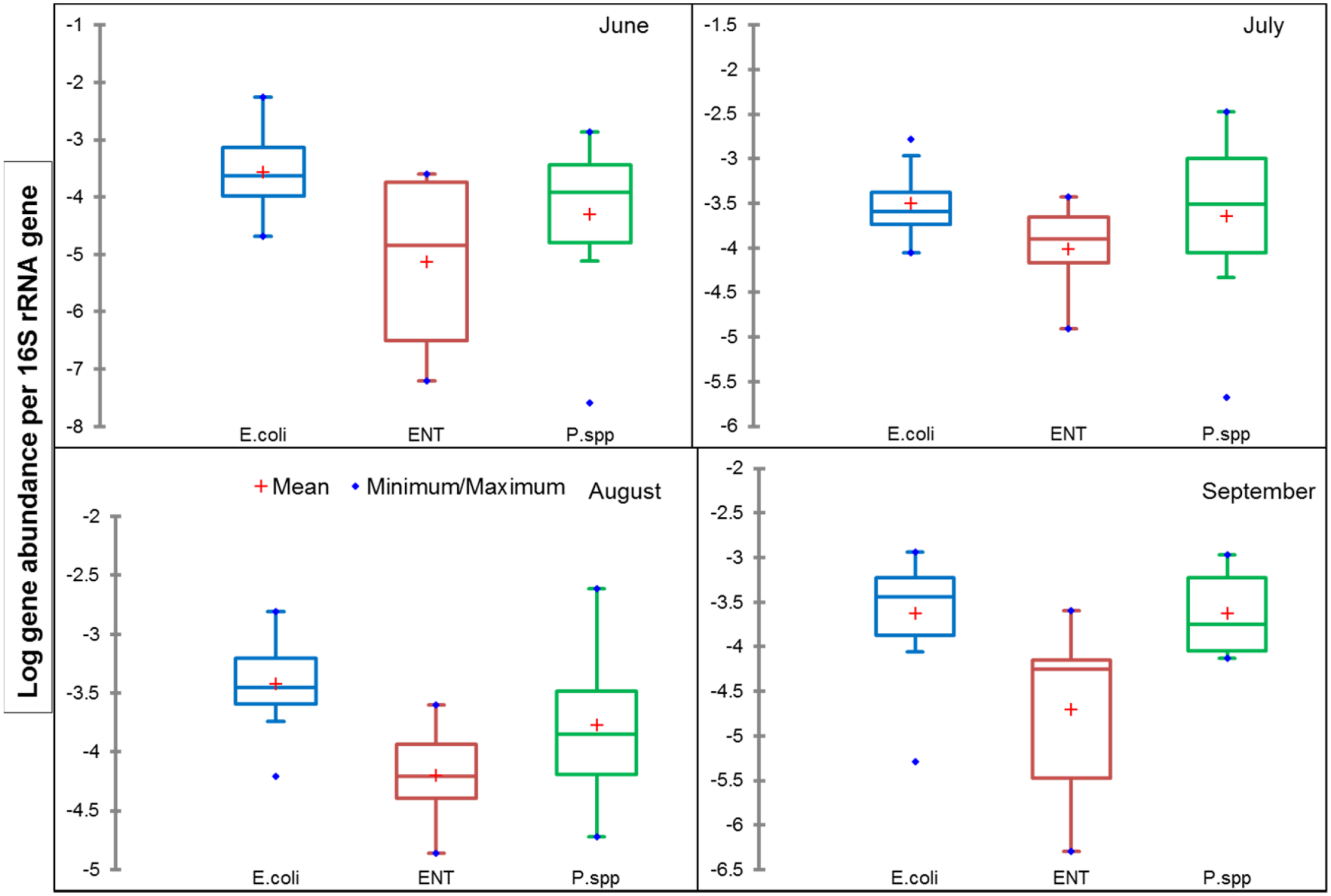
Gene copy number of selected bacterial species (*E*. *coli*, ENT, and *Pseudomonas* spp.) normalized to 16S rRNA gene copy numbers in sediment samples collected for 4 months (June to September) at selected HOP. The box plot represents the first and third quartile, the blue dots on maximum/minimum values and the red cross indicate mean.

**Fig 3:**
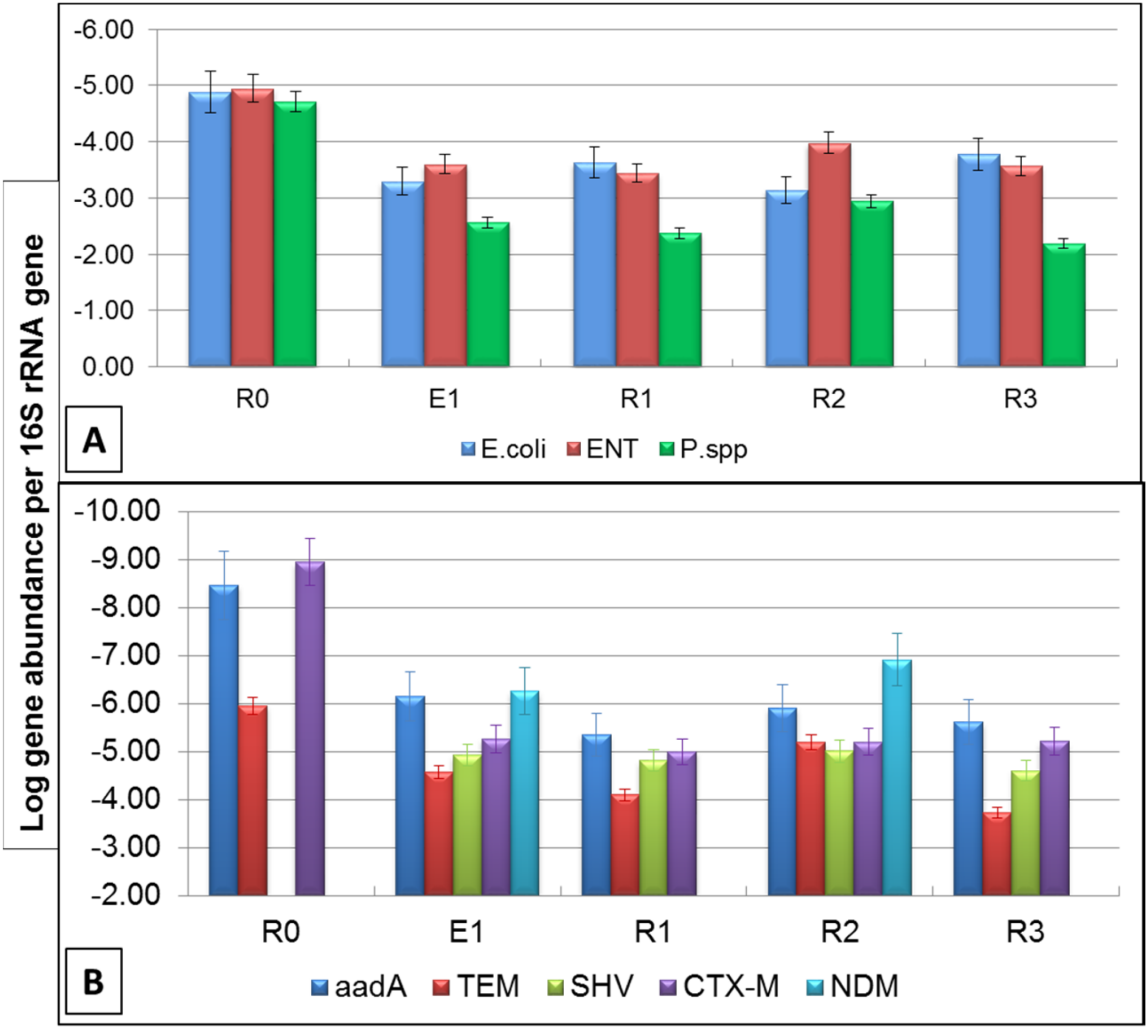
Gene copy number of (A) selected bacterial species (*E*. *coli*, ENT, and *Pseudomonas* spp.) and (B) selected antibiotic resistance genes (*aadA*, *bla*_TEM_, *bla*_CTX‑M_, *bla*_SHV_, and *bla*_NDM_) normalized to 16S rRNA gene copy numbers in the sediment samples collected in September 2013 from CRB, Tiruchirappalli, Tamil Nadu, India.

The average gene copy numbers of the bacterial marker genes for *E*. *coli*, ENT and *Pseudomonas* spp. in the sediment samples are presented in Figures A (top panel) and B in S1 File. The bacterial density at HOP varied considerably; ranging from 5.9 to 8.5, 4.3 to 7.9, and 4.0 to 8.9 16S rRNA gene normalized log copy numbers for *E*. *coli*, ENT and *Pseudomonas* spp., respectively (Fig 2). A similar degree of variation was observed in the sediment samples from CRB (Fig 3A). The HOP site had 120, 104 and 89 fold higher bacterial load for *E*. *coli*, ENT and *Pseudomonas* spp. respectively than CRB samples. At CRB the abundance for the selected marker genes was generally low, except for the E1 site. Compared to the other sampling points on the river, E1 had higher abundance values of -3.3, -3.6 and -2.5 log values for *E*. *coli*, ENT and *Pseudomonas* spp., respectively, which could possibly be influenced by the discharge of untreated urban and hospital effluents. Effluent discharge had no influence at site R0 (the control site in this study), located upstream of E1. Hence the abundance levels for the selected bacterial marker genes were relatively low. Studies have suggested that ratio proportion of E. coli to ENT can be used to identify the relative presence of human and animal faeces [24]. The ratio of E. coli/ENT is higher in human faeces than animal sources of fecal contamination in the aquatic systems and composition of fecal flora differ significantly faeces [25]. In this study, the E. coli/ENT ratios estimated for CRB and HOP were 1.19 and 5.09, which is in complete agreement with some previous studies [24, 25].

Environments such as water, soil and sediment have previously shown to represent a secondary habitat for FIB (*E*. *coli* and ENT) outside the gastrointestinal tract of warm blooded animals. Recent studies reported that *E*. *coli* can reproduce and persist in these secondary environments in both temperate and tropical climates [6, 26, 27]. The presence of FIB in these secondary environments is used as an indicator of fecal pollution [6]. Sediments provide favorable conditions (temperature, nutrients) for growth and proliferation; shield them from sunlight inactivation and protozoan gazing [6, 7, 11, 26]. Hence, it is evident that sediment in the effluent receiving system could act as a potential reservoir of bacterial populations from human and animal sources. Resuspension of these contaminants accumulated in the sediment could affect the water quality and pose a threat to human and animal health.

### Antibiotic resistance genes in sediments

**Fig 4:**
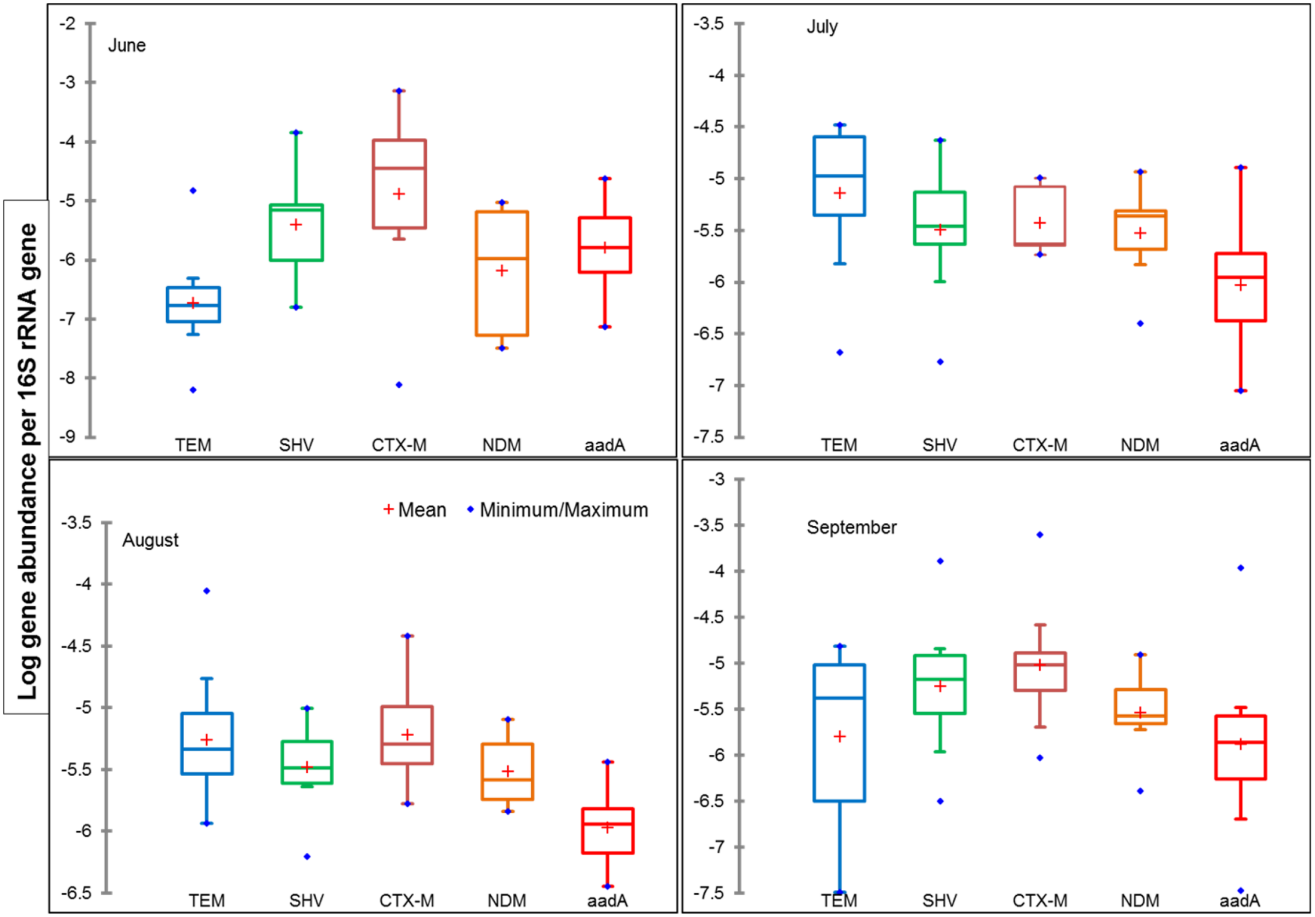
Gene copy numbers of antibiotic resistance genes (*bla*_TEM_, *bla*_CTX‑M_, *bla*_SHV_, *bla*_NDM_ and *aadA*,) normalized to 16S rRNA gene copy numbers in sediment samples collected for 4 months (June to September) at selected HOP. The box plot represents the first and third quartile, the blue dots on maximum/minimum values and the red cross indicate mean.

The gene copy numbers of selected ARGs from CRB and HOP were quantified by qPCR and the results are presented in Figures A (lower panel) and C in S1 file. The ARGs conferring resistance to ß-lactams were selected for the study based on the criteria that two-third of Abs administered to humans are ß-lactams [28]. The gene copy numbers at CRB varied from 4.7–6.1, 0–6.4, 1.7–6.1, 3.5–5.1 and 2.2–5.2 16S rRNA gene normalized log copy numbers for *bla*_TEM_, *bla*_SHV_, *bla*_CTX-M_, *bla*_NDM_ and *aadA*, respectively. Normalizing the ARGs and calculating for gene ratios serves as a proxy for the proportion of bacteria carrying ARGs [29]. In this study we use 16S rRNA gene normalized data for CRB and HOP samples (Figs 2B and 4). Assuming the ARGs and 16S rRNA gene extractions were analogous, normalizing with 16S rRNA gene supported for minor variations in DNA extraction efficiency. Additionally, the relative abundances of the studied ARGs provide a possibility to compare data among ARGs quantified in various other studies. In a previous study [30], *bla*_TEM_ was one of the most frequently detected plasmid-borne genes, which confers resistance to penicillin's and extended spectrum Cephalosporin's. High prevalence of *bla*_TEM_ and *aadA* are also reported in the periodic sediments of Lake Geneva, from the start of twentieth century [11, 12] supporting the fact that these genes were carried by bacteria as structural integrated reservoirs even before the inventions of Abs [31]. Similarly, among sediment samples analyzed in this study, the ARGs *bla*_TEM_ and *aadA* were identified at all sampling sites with relative abundances ranging from -6.8 to -3.7 and -8.4 to -4.5 16S rRNA gene normalized log copy numbers, respectively. These results emphasize that the ARGs like *bla*_TEM_ are likely to be endemic in the terrestrial environment [17, 32].

Studies have highlighted that the amount of ARGs in the sediments close to urban settlements is much higher than in pristine environments [11, 12, 33]. In this study, *bla*_SHV_ and *bla*_NDM_ (metallo ß-lactamase—MBL) were absent in the control sample (R0) but identified in samples taken downstream of the effluent discharge point in CRB. Also the relative abundance of extended spectrum ß-lactamases (ESBLs) like *bla*_CTX-M_ was higher in sediment samples collected after the effluent discharge point than at the control site. These results clearly indicate a role for urban and hospital effluents in the dissemination of ARGs to the aquatic environment. The prevalence of microbial contaminants (e.g. *bla*_CTX-M_) at the control site could be explained by the input from major activities like agricultural runoff, sand dredging, religious rituals, open defecation, urban discharges and other anthropogenic activities in the upper River Cauvery, which receives considerable amount of wastewaters on its course to Bay of Bengal [22, 23].

Higher levels of ARGs were observed in the sediments of HOP than those in the CRB. In a comparison between the CRB and HOP, the ARGs abundances were ca. 49.7, 50.9, 39.5, 32.8 and 40.3 fold higher for *bla*_TEM_, *bla*_CTX-M_, *bla*_SHV_, *bla*_NDM_ and *aadA*, respectively at HOP. Most of the ARGs had a seasonal variation at HOP. These variations could be explained by the type of hospitals, sampling period and the disposal practices at the study sites, which all play a major role in the dissemination of clinically relevant ARGs. The *bla*_CTX-M_ gene is currently the most common ESBL worldwide, with CTX-M-15 and CTX-M-14 predominantly invading humans and animals as well as environmental compartments [34]. The spread of ESBL and MBL in the freshwater receiving system is highly alarming. The majority of ESBL/MBL producing strains are also resistant to other clinically relevant Abs. This is due to the fact that these ß-lactamases are commonly carried on conjugative plasmids that also harbor gene conferring resistance to other antibiotic classes [35]. The presence of ESBLs/MBLs genes in the freshwater sources are also reported in other countries, including France, Portugal, Finland, the United States, Brazil, Switzerland, China and Pakistan [8, 11, 19, 32, 35–37], indicating that a global environmental dissemination of ARGs is currently taking place.

### Correlation between microbial indicators

**Fig 5:**
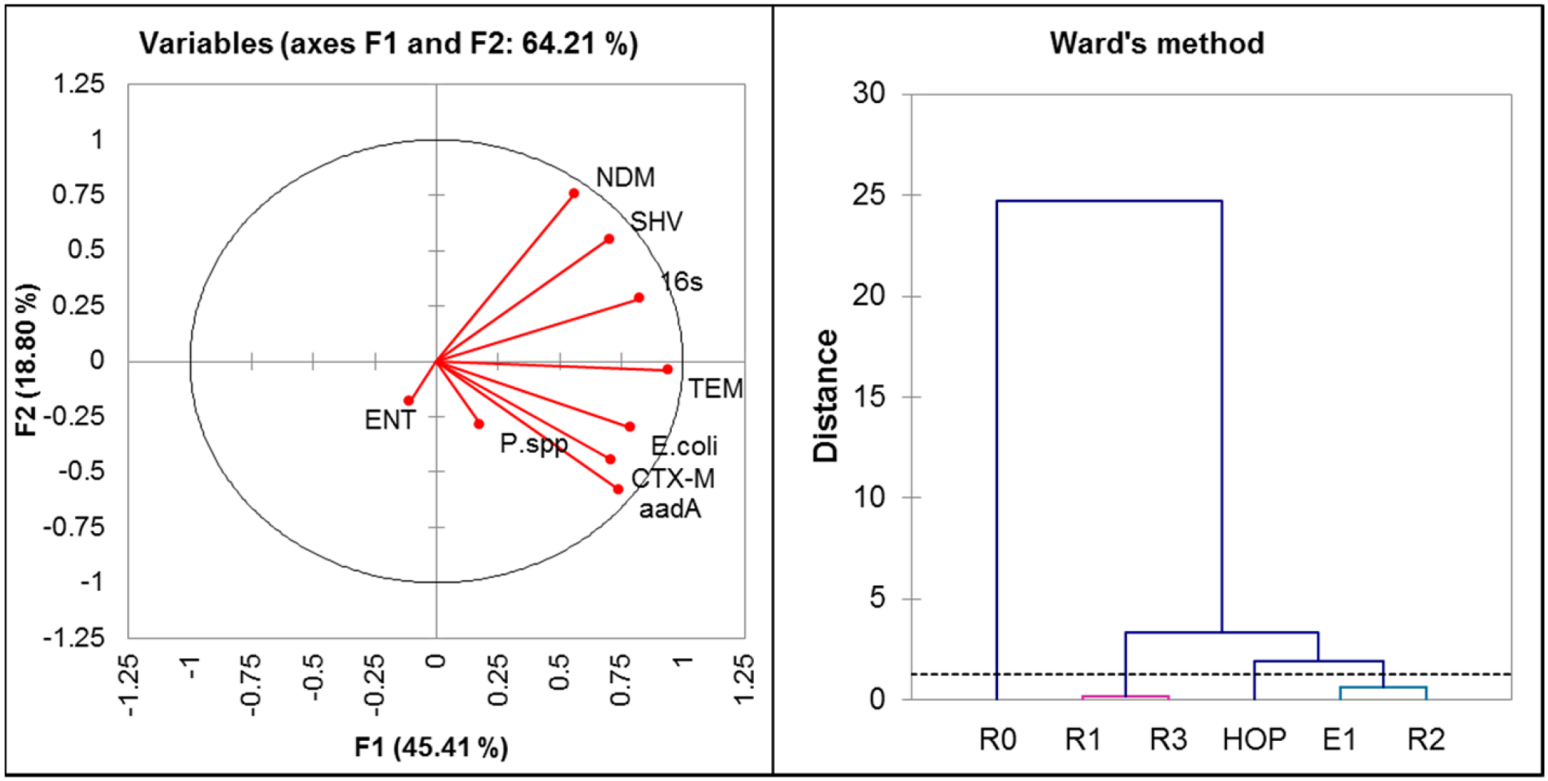
Principle Component Analysis (PCA), on correlation biplot (left panel) indicating the correlations among studied variables and biplot scores on axis 1 and 2 are representing 64.21%. Agglomerative hierarchical clustering (AHC) computation based on Ward's method (right panel).

Inspection of Fig 5 (right panel) shows that the contaminated sites at CRB (E1, R1-R3) are closely grouped with the HOP, and separately clustered to the control site at a greater distance, substantiating the high prevalence of ARGs and bacterial indicators at the HOP and downstream of the effluent discharge point in CRB. The positive mutual correlation [Fig 5 (left panel) and Table B in S1 file] of all of the ARGs is significant, as well as the correlation of ARGs with total bacterial load (16S rRNA gene); correlation coefficients ranged from 0.3 to 0.7 (*p* < 0.05, n = 45). Likewise *E*. *coli* also demonstrated a positive correlation (r > 0.3, *p* < 0.05, n = 45) with ARGs (except NDM). The bacterial species, ENT and *Pseudomonas* spp. were positively correlated (r = 0.5) with each other. The *aadA* gene, frequently identified on the mobile genetic elements [38] (class 1 integron genes) had a positive correlation (r > 0.4, *p* < 0.05, n = 45) with 16S rRNA gene, *E*. *coli* and ARGs (*bla*_*TEM*_ and *bla*_*CTX-M*_). This implies a ß-lactamases association with these mobile genetic elements. This is also supported by a recent study on untreated hospital wastewaters in Brazil, reporting on high prevalence of ß-lactamases and class 1 integron genes (41.9%) [8]. Positive correlation between 16S rRNA gene, *E*.*coli* and studied ARGs emphasizes that these contaminants stem from a common and contemporary source. This results in environments where ARGs, resistant bacteria and the environmental bacterial flora (which harbors ARGs) are mixed and play a major role in the dissemination of antibiotic resistance to the aquatic environment. Environment of these types are likely to act as resistance hotspots where they support ARGs proliferation and the emergence of new resistance strains [33, 36]. Recent metagenomic studies aimed at understanding microbial populations in clinical environments highlight the differences in bacterial communities invarious sections of the hospital [39] and in the sediments receiving urban effluents [40]. This observation warrants further studies to understand the path and processes of selection of AR dissemination in the bacterial communities, and which are required to reduce the risks associated with human and animal health.

The emergence and spread of antibiotic resistance are complex processes with many factors affecting co-selection and cross-selection of resistance. Metals (such as Cd, Cu, Hg and Zn) can selectively induce the co-selection of antibiotic resistance if they spread and accumulate in the environment at critical concentrations [41]. From our previous study [13] it is evident that the metal concentration in the studied locations (CRB/HOP) are above the probable effect concentrations as recommended by the sediment quality guidelines [42]. Horizontal gene transfer (HGT) mechanisms between bacterial hosts are also considered as key factors behind the elevated concentrations of ARGs in the environment [1, 43]. A recent study [18] reported that the rate of gene mobilization at 30°C is higher than that at 37°C. Hence metal concentrations and climatic factors may contribute to elevated concentrations of ARGs at our tropical study site. Existence and proliferation of antibiotic resistance in pathogenic and zoonotic bacteria has strong associations with human/animal health as these bacteria could be transmitted via direct or indirect contact. The acquisition of resistance genes by bacteria (esp. ESBLs, MBLs) minimizes the therapeutic options and probably lead to frequent infections [28].

Molecular signature studies on ARGs from pristine and highly polluted sites provide evidence that transport of contaminants (ARGs) from the source is also an important factor for the dissemination of ARGs to the aquatic ecosystem [44, 45]. Molecules such as DNA are capable of interacting with the surfaces in complex ways, including multiple sorption methods [46, 47]. Sorbed to flocs, suspended solids, and/or organic matter these ARB/ARGs could be transported along with the river currents [48]. In this study, the clinically relevant ARGs (ESBLs and MBLs) were detected at a distance of ca. 15 km from the effluent discharge point. This sample site (R3) “The Grant Anicut” (reservoir) serves in the Cauvery Delta as a major source of irrigational water. While surface water contamination and cross contamination of irrigational water are suspected for some large outbreaks, increasing evidence of human gastrointestinal illness due to potential contamination by pathogenic microbes in fresh produce have been reported [49]. Nevertheless, one may argue that studies that analyze the DNA such as this study, while providing information on the presence/absence or even quantitative data but do not provide information on the expression of these ARGs [50]. However, the expression of genes is not the central query of this study when the purpose of this study is to address the evaluation of the tropical aquatic environment to serve as reservoirs of ARGs that could be potentially transferred to other bacterial cells through HGT.

In most developing nations rivers are not only an important part of the country’s economy but also can act as a dumping site for communities and industries situated along the river. Wastewater discharges from various sources (industries, hospitals, and communities) have a direct influence on the proliferation and dissemination of emerging contaminants along the receiving river system. Overall, this study supports the hypothesis that the natural environment (i.e., sediments) can act as a reservoir for emerging microbial contaminants such as FIB and ARGs. The relative abundance of ARGs vary according to the seasonal sampling period at HOP and it is likely that these ARGs released/accumulated in the sediments of the sewer systems could be transported to the receiving systems in large quantities during floods and periods of rain. Sediments receiving wastewaters from contemporary sources offer the opportunity to restructure the pollution history and allow us to evaluate the potential impacts of pollution [11]. The quantification of ARGs in the aquatic ecosystem will facilitate improved risk assessments for the prudent use of Abs in human, animal and agriculture, and will provide baseline information for developing strategies to limit the spread of antibiotic resistance. The results of this study suggest that the discharge of hospital and urban wastewaters into the aquatic systems leads to the dissemination of emerging microbial pollutants. Therefore, the river receiving systems under tropical conditions such as CRB which has average daily peak temperatures reaching 30°C, could potentially favor the transfer of mobile genetic elements carrying ARGs to susceptible bacterial pathogens. Hence further studies are required to unravel the pathways involved in the spread of ARGs and FIB for assessing the human and environment potential risks in similar geographical locations.

## Supporting Information

S1 File This file includes Tables A and B; Figures A, B and C. (PDF)
