# Supplementary material for: Occurrence of Antibiotic Resistance Genes and Bacterial Markers in a Tropical River Receiving Hospital and Urban Wastewaters
Source: PLoS One. 2016 Feb 24;11(2):e0149211. doi: 10.1371/journal.pone.0149211 (PMC4766091; doi:10.1371/journal.pone.0149211)
Supplement: S1 File — (PDF) [file pone.0149211.s001.pdf]

Occurrence of antibiotic resistance genes and bacterial markers in a tropical river receiving hospital and urban effluent waters

Naresh Devarajan[1], Amandine Laffite[1], Crispin Kyela Mulaji[2], Jean-Paul Otamonga[3], Pius Tshimankinda Mpiana[2], Josué Ilunga Mubedi[3], Kandasamy Prabakar[4], Bastiaan Willem Ibelings[1], John Poté[1,2,3*]

[1]*University of Geneva, Faculty of Sciences, Earth and Environmental Sciences, Institute F. A. Forel and Institute of Environmental Sciences, Bd Carl-Vogt 66, CH-1211 Geneva 4, Switzerland*

[2]*University of Kinshasa (UNIKIN), Faculty of Science, Department of Chemistry, B.P. 190, Kinshasa XI, Democratic Republic of the Congo*

[3]*Université Pédagogique Nationale (UPN). Croisement Route de Matadi et Avenue de la Libération. Quartier Binza/UPN, B.P. 8815 Kinshasa, Democratic Republic of the Congo*

[4]*Postgraduate and Research Department of Zoology, Jamal Mohamed College, Tiruchirappalli-620020, Tamil Nadu, India.*

Supporting Information (S1 file)

No of Tables = 2 (Table A and B)

No of Figures = 3 (Figure A, B and C)

Table A. Primers used for plasmid contruction and quantification with RT-PCR

Gradient PCR for plasmid construction					
Target organism/gene	Primer	Sequence (5' – 3')	Tm (˚C)	Size (bp)	Referen ce
E. coli (uidA)	298F	AATAATCAGGAAGTGATGGA GCA	53	587	1
	884R	CGACCAAAGCCAGTAAAGTA GAA			
ENT (16S rRNA gene)	ENT-151-F	ACACTTGGAAACAGGTGC	53	458	2
	Ent-578-R	TTAAGAAACCGCCTGCGC			
Pseudomonas spp. (16S rRNA gene)	PAGS-F	GACGGGTGAGTAATGCCTA	54	618	3
	PAGS-R	CACTGGTGTTCCTTCCTATA			
$bla_{CTX\text{-}M}$	blaCTX-M-f	CGCTTTGCGATGTGCAG	52	593	4
	blaCTX-M-r	ACCGCGATATCGTTGGT			
bla_{SHV}	blaSHV F	ATGCGTTATATTCGCCTGTG	56	865	5
	blaSHV R	GTTAGCGTTGCCAGTGCTCG			
$bla_{NDM\text{-}1}$	NDM-Fm	GGTTTGGCGATCTGGTTTTC	53	621	6
	NDM-Rm	CGGAATGGCTCATCACGATC			
qPCR for quantification					
Bacterial V3 region	338 F	ACTCCTACGGGAGGCAGCAG	55	197	7
	518 R	ATTACCGCGGCTGCTGG			
E. coli (uidA)	Uida 405 F	CAACGAACTGAACTGGCAGA	55	121	8
	Uida 405 R	CATTACGCTGCGATGGAT			
ENT (16S rDNA)	Ent376F	GGACGMAAGTCTGACCGA	55	221	1

	Ent578R	TTAAGAAACCGCCTGCGC			
Pseudomonas spp. (16S rDNA)	Pse435F	ACTTTAAGTTGGGAGGAAGGG	55	251	9
	Pse686R	ACACAGGAAATTCCACCACCC			
bla_{TEM}	TEM-RT-F	GCKGCCAACTTACTTCTGACAACG	55	247	10
	TEM-RT-R	CTTTATCCGCCTCCATCCAGTCTA			
bla_{CTX-M}	blaCTX-M-rt-f	ATTCCRGGCGAYCCGCGTGATACC	62	227	11
	blaCTX-M-rt-r	ACCGCGATATCGTTGGTGGTGCCAT			
bla_{SHV}	blaSHV-rt-f	CGCTTTCCCATGATGAGCACCTTT	60	110	12
	blaSHV-rt-r	TCCTGCTGGCGATAGTGGATCTTT			
bla_{NDM-1}	NDM-F	TTGGCGATCTGGTTTTCC	58	195	13
	NDM-R	GGTTGATCTCCTGCTTGA			
aadA	aadA-F	GCAGCGCAATGACATTCTTG	55	282	14
	aadA-R	ATCCTTCGGCGCGATTTTG			

Table B. Correlation matrix (Pearson) performed for the analyzed parameters[a] in the surface sediment samples.

Variables	16s	*E.coli*	ENT	P.spp	*aadA*	TEM	SHV	CTX-M	NDM
16s	**1**	**0.529**	-0.090	0.248	**0.442**	**0.718**	**0.625**	**0.365**	**0.675**
E.coli		**1**	0.032	0.176	**0.686**	**0.722**	**0.362**	**0.565**	0.233
ENT			**1**	**0.555**	-0.069	-0.156	-0.107	-0.163	0.011
P.spp				**1**	0.145	0.146	-0.008	0.032	-0.008
aadA					**1**	**0.730**	0.198	**0.758**	-0.046
TEM						**1**	**0.611**	**0.653**	**0.492**
SHV							**1**	**0.301**	**0.749**
CTX-M								**1**	0.047
NDM									**1**

[a] Parameters include bacterial population (16s rRNA gene, *E. coli*, ENT, *Pseudomonas* spp. (P.spp), and the selected ARGs (*aadA*, bla_{TEM}, bla_{SHV}, $bla_{\mathrm{CTX\text{-}M}}$, and bla_{NDM}). Correlation coefficients have been calculated using the log transformed values to normalize their distribution. Statistically significant coefficients ($p < 0.05$) are in bold.

Figure A. qPCR quantification of (A) for the selected bacterial species (*E. coli*, ENT and *Pseudomonas* spp.) and (B) antibiotic resistance genes (*aadA*, bla_{TEM}, bla_{SHV} $bla_{\text{CTX-M}}$ and bla_{NDM}) in the surface sediments of Cauvery River Basin. Values are expressed as copy numbers per gram of dry sediment.

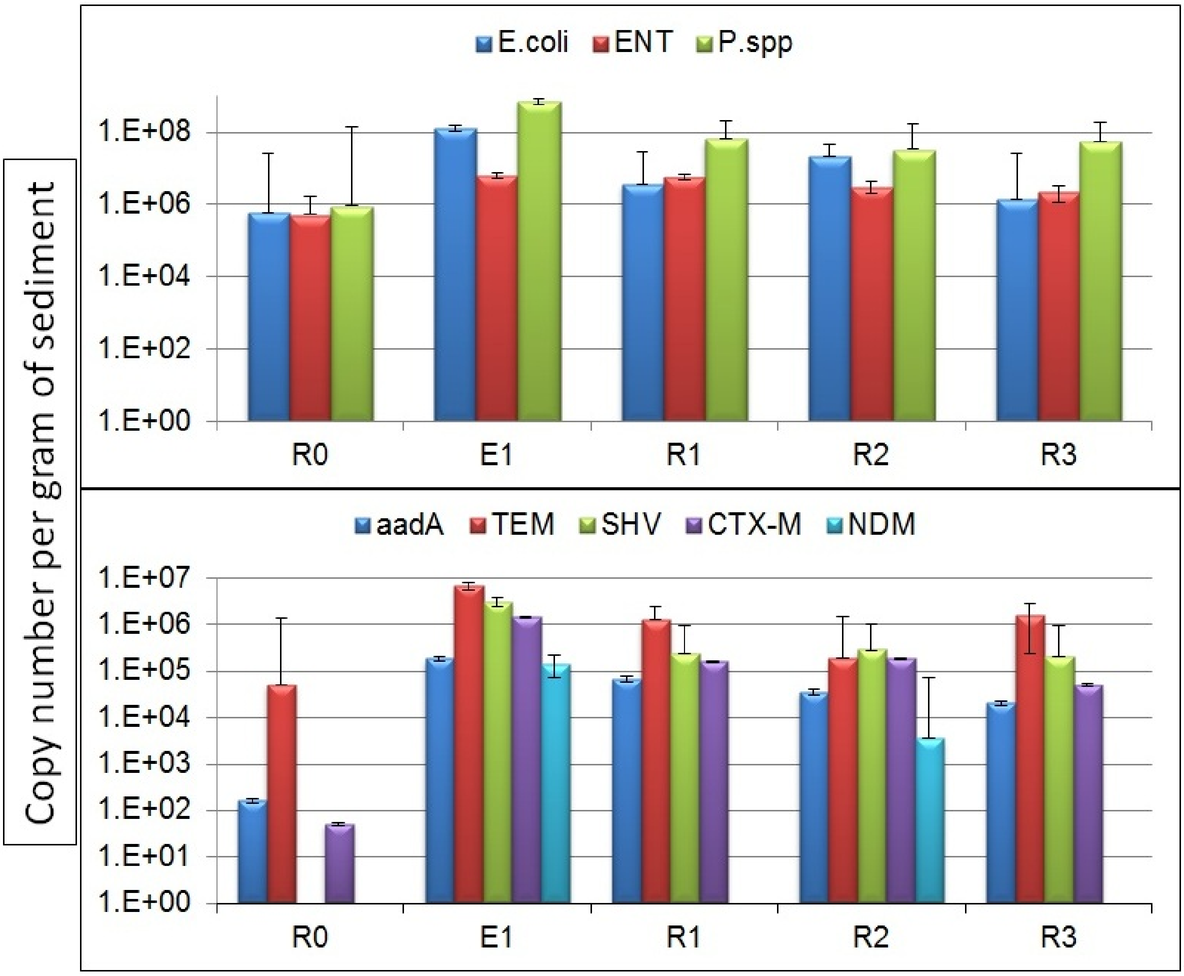

Figure B. qPCR quantification of the bacterial population for the selected bacterial groups (*E. coli*, ENT and *Pseudomonas* spp.) in the surface sediments of HOP samples. Values are expressed as copy numbers in per gram of dry sediment. The box plot represents the first and third quartile, the blue dots on maximum/minimum values and the red cross indicate standard errors

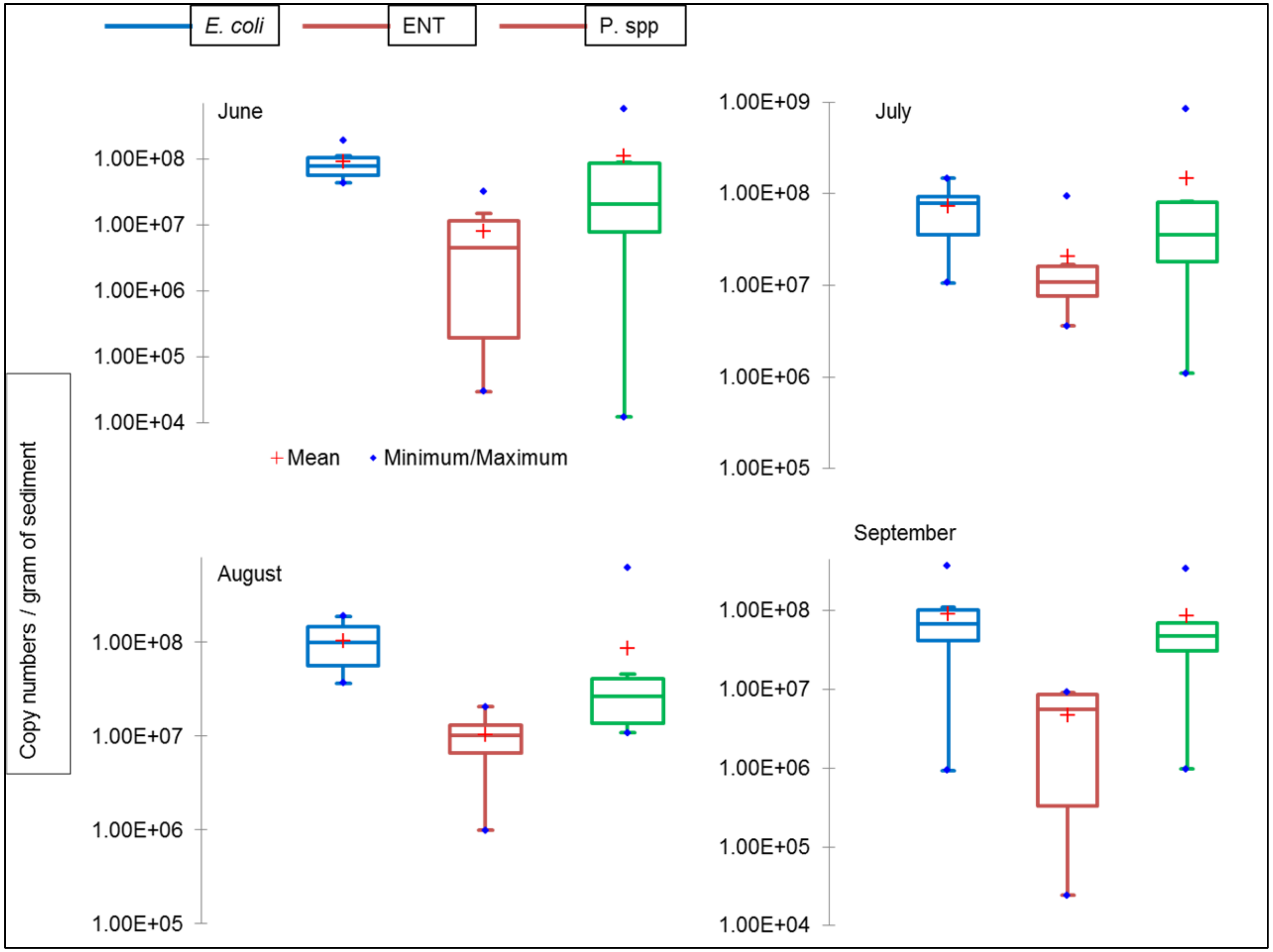

Figure C. qPCR quantification of the selected antibiotic resistance genes (*bla*$_{TEM}$, *bla*$_{SHV}$, *bla*$_{CTX-M}$, *bla*$_{NDM}$ and *aadA*) in the surface sediments of HOP samples. Values are expressed as copy numbers in per gram of dry sediment. The box plot represents the first and third quartile, the blue dots on maximum/minimum values and the red cross indicate standard errors

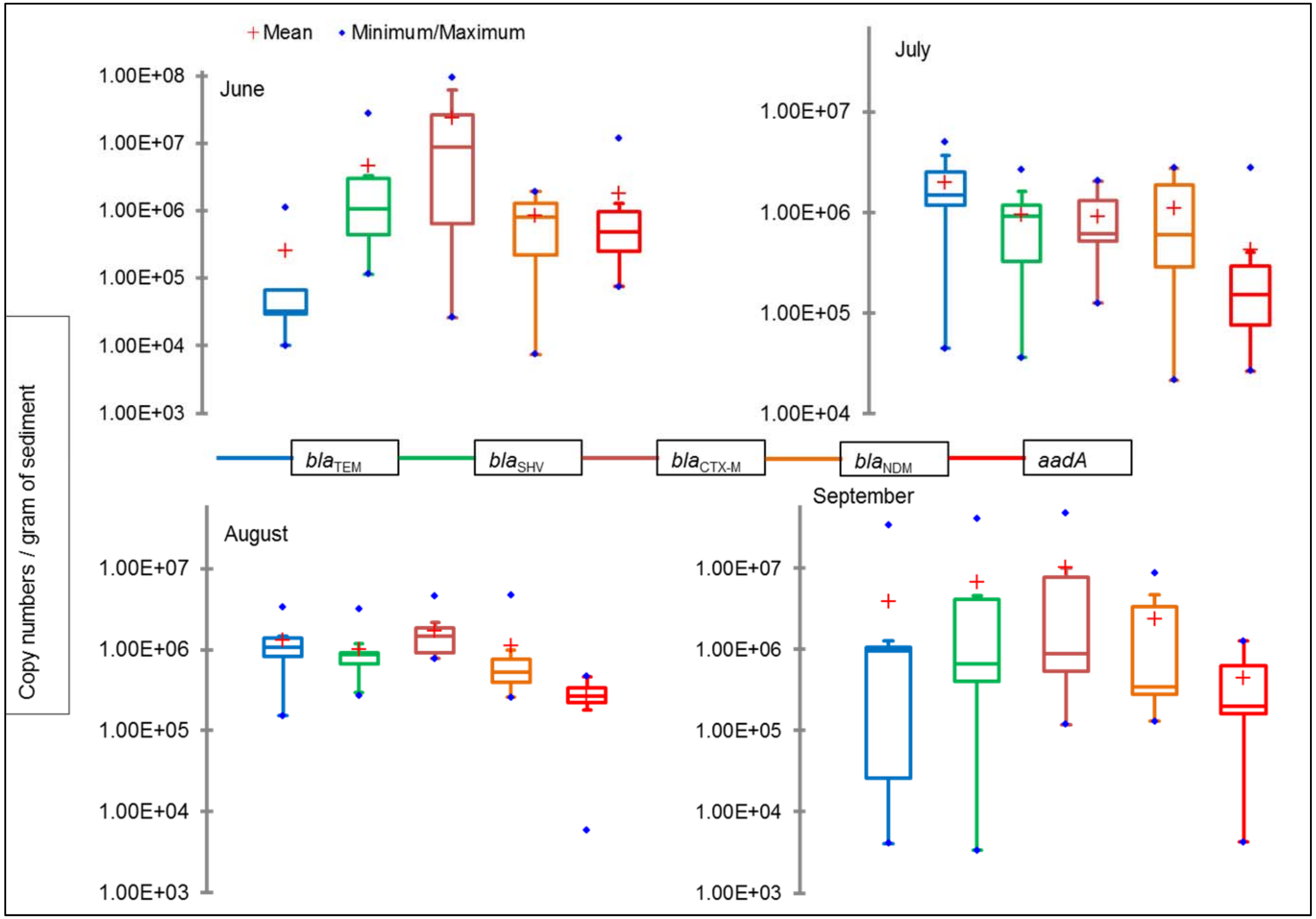

S1 References

1. Ram, J. L.; Ritchie, R. P.; Fang, J.; Gonzales, F. S.; Selegean, J. P. Sequence-Based Source Tracking of Based on Genetic Diversity of β-Glucuronidase. *Journal of environmental quality*. **2004**, *33(3)*, 1024-1032.
2. Ryu, H.; Henson, M.; Elk, M.; Toledo-Hernandez, C.; Griffith, J.; Blackwood, D.; Noble, R.; Gourmelon, M.; Glassmeyer, S.; Santo Domingo, J. W. Development of quantitative PCR assays targeting the 16S rRNA genes of Enterococcus spp. and their application to the identification of Enterococcus species in environmental samples. *Appl. Environ. Microbiol*. **2013**, *79 (1)*, 196–204.
3. Spilker, T., Coenye, T., Vandamme, P., LiPuma, J. J. PCR-Based assay for differentiation of Pseudomonas aeruginosa from other Pseudomonas species recovered from cystic fibrosis patients. *J Clin Microbiol*. **2004**, *42*, 2074–2079.
4. Bonnet, R.; Dutour, C.; Sampaio, J. L. M.; Chanal, C.; Sirot, D.; Labia, R.; De Champs, C.; Siroti, J. Novel Cefotaximase (CTX-M-16) with Increased Catalytic Efficiency Due to Substitution Asp-2403 Gly. *Antimicrobial agents and chemotherapy*. **2001**, *45*, 2269-2275.
5. Paterson, D. L.; Hujer, K. M.; Hujer, A. M.; Yeiser, B.; Bonomo, M. D.; Rice, L. B.; Bonomo, R. A. Extended-spectrum β-lactamases in Klebsiella pneumoniae bloodstream isolates from seven countries: dominance and widespread prevalence of SHV-and CTX-M-type β-lactamases. *Antimicrobial agents and chemotherapy*. **2003**, *47*(11), 3554-3560.
6. Nordmann, P.; Poirel, L.; Carrër, A.; Toleman, M. A.; Walsh, T. R. How to detect NDM-1 producers. *Journal of clinical microbiology*, **2011**, *49*(2), 718-721.
7. Ovreas, L.; Forney, L.; Daae, F. L.; Torsvik, V. Distribution of bacterioplankton in meromictic Lake Saelenvannet, as determined by denaturing gradient gel electrophoresis of PCR-amplified gene fragments coding for 16s rRNA. *Applied and Environmental Microbiology*. **1997**, *63*:3367-3373.
8. Chern, E. C.; Siefring, S.; Paar, J.; Doolittle, M.; Haugland, R. A. Comparison of quantitative PCR assays for Escherichia coli targeting ribosomal RNA and single copy genes. *Letters in applied microbiology*. **2011**, *52*(3), 298-306.

9. Bergmark, L.; Poulsen, P. H. B.; Al-Soud, W. A.; Norman, A.; Hansen, L. H.; Sørensen S. J. Assessment of the specificity of Burkholderia andPseudomonas qPCR assays for detection of these genera in soil using 454 pyrosequencing. *FEMS. Micrbio Let*. **2012**, DOI: http://dx.doi.org/10.1111/j.1574-6968.2012.02601.x 77-84
10. Sidrach-Cardona, R.; Hijosa-Valsero, M.; Marti, E.; Balcázar, J. L.; Becares, E. Prevalence of antibiotic-resistant fecal bacteria in a river impacted by both an antibiotic production plant and urban treated discharges. *Science of the Total Environment*. **2014**, *488–489*, 220–227.
11. Fujita, S. I.; Yosizaki, K.; Ogushi, T.; Uechi, K.; Takemori, Y.; Senda, Y. Rapid identification of Gram-negative bacteria with and without CTX-M extended-spectrum β-lactamase from positive blood culture bottles by PCR followed by microchip gel electrophoresis. *Journal of clinical microbiology*. **2011**, *49*(4), 1483-1488.
12. Xi, C.; Zhang, Y.; Marrs, C. F.; Ye, W.; Simon, C.; Foxman, B.; Nriagu, J. Prevalence of antibiotic resistance in drinking water treatment and distribution systems. *Applied and environmental microbiology*. **2009**, *75*(17), 5714-5718.
13. Zheng, F.; Sun, J.; Cheng, C.; Rui, Y. The establishment of a duplex real-time PCR assay for rapid and simultaneous detection of bla NDM and bla KPC genes in bacteria. *Annals of clinical microbiology and antimicrobials*. **2013**, *12*(1), 30.
14. Madsen, L.; Aarestrup, F. M.; Olsen, J. E.Characterization of Streptomycin Resistance determinants in Danisk isolates of Salmonella typhimurium. *Vet. Microbiol*. **2000**, *75*, 73-82.
